# Ginsenoside Rh1 Prevents Migration and Invasion through Mitochondrial ROS-Mediated Inhibition of STAT3/NF-κB Signaling in MDA-MB-231 Cells

**DOI:** 10.3390/ijms221910458

**Published:** 2021-09-28

**Authors:** Yujin Jin, Diem Thi Ngoc Huynh, Chang-Seon Myung, Kyung-Sun Heo

**Affiliations:** College of Pharmacy and Institute of Drug Research and Development, Chungnam National University, Daejeon 34134, Korea; 201850535@o.cnu.ac.kr (Y.J.); ngocdiemphar@gmail.com (D.T.N.H.); cm8r@cnu.ac.kr (C.-S.M.)

**Keywords:** metastasis, ginsenoside Rh1, mitochondrial ROS, STAT3, NF-κB, triple-negative breast cancer cells

## Abstract

Breast cancer (BC) a very common cancer in women worldwide. Triple negative breast cancer (TNBC) has been shown to have a poor prognosis with a high level of tumor metastatic spread. Here, the inhibitory effects of ginsenoside-Rh1 (Rh1) on BC metastasis, and its underlying signaling pathway in TNBC were investigated. Rh1-treated MDA-MB-231 cells were analyzed for metastasis using a wound healing assay, transwell migration and invasion assay, western blotting, and qRT-PCR. Rh1 treatment significantly inhibited BC metastasis by inhibiting the both protein and mRNA levels of MMP2, MMP9, and VEGF-A. Further, Rh1-mediated inhibitory effect on BC migration was associated with mitochondrial ROS generation. Rh1 treatment significantly eliminated STAT3 phosphorylation and NF-κB transactivation to downregulate metastatic factors, such as MMP2, MMP9, and VEGF-A. In addition, Mito-TEMPO treatment reversed Rh1 effects on the activation of STAT3, NF-κB, and their transcriptional targets. Rh1 further enhanced the inhibitory effects of STAT3 or NF-κB specific inhibitor, stattic or BAY 11-7082 on MMP2, MMP9, and VEGF-A expression, respectively. In summary, our results revealed the potent anticancer effect of Rh1 on TNBC migration and invasion through mtROS-mediated inhibition of STAT3 and NF-κB signaling.

## 1. Introduction

Breast cancer (BC) is the leading cause of cancer-related death among women worldwide [1]. BC is heterogeneous cancer that is divided into four types of cancer depending on receptor expression [2]. Among them, triple-negative breast cancer (TNBC) is an aggressive phenotype that has a poor prognosis, and has therapeutic limitations due to metastasis, epithelial mesenchymal transition, chemodrug-resistance, and gene mutations [3,4]. Metastasis is the primary cause of cancer recurrence and invades other tissues through the blood circulation [5]. Molecular mechanisms of metastasis start with the expression of matrix metalloproteinases (MMPs) and angiogenesis [6]. Angiogenesis is a critical cascade of tumor development, migration, and invasion [6]. Many researchers have attempted to develop new drugs or therapeutic trials targeting the receptor or signaling pathway to inhibit TNBC metastasis. However, therapeutic trials have limitations because of cell turns on other signaling pathways or gene mutations to escape the therapeutic effects [5].

Reactive oxygen species (ROS) are secondary messengers contributing to various signaling pathways, including proliferation and metastasis in cancer [7]. Previous reports demonstrated that ROS can regulate cancer proliferation and apoptosis by ROS-mediated kinase activation, and its effects were inhibited by ROS scavengers, such as N-acetyl-cysteine (NAC) [8,9]. Mitochondrial ROS (mtROS) contributes to the regulation of mitochondrial mechanisms involved in cancer homeostasis and development [7]. It has been reported that ROS contributes to BC metastasis by induction of phosphatidylinositol-3-kinase/protein kinase B (PI3K/Akt) or ROS-mediated mitogen-activated protein kinase (MAPK) signaling pathway [10,11].

It has also been reported that metastasis is regulated by various signaling pathways such as MAPK, PI3K/Akt, signal transducer and activator of transcription (STAT), and nuclear factor–kappa B (NF-κB) pathways [9,12,13,14]. Among them, STAT3, a member of the STAT family, functions as a transcription factor that regulates inflammation, tumorigenesis, angiogenesis, and stemness [15]. Several studies have demonstrated that STAT3 plays a crucial role in BC proliferation and metastasis by inducing MMP family proteins such as MMP2, 7, 9, and 13 or vascular endothelial growth factor (VEGF) expression through gene regulation [9,14,15]. NF-κB is composed of p65 and p50, and phosphorylated p65 and p50 can function as transcription factors. Besides, NF-κB is an inflammation-related kinase that increases expression of interleukin-1, interleukin-6, tumor necrosis factor-α, and MMPs by the NF-κB-dependent signaling pathway [16].

Ginsenosides are extracted from saponin, which is divided into 20(S)-protopanaxadiol saponin and 20(S)-protopanaxatriol saponins (PTS), depending on the molecular structure and metabolic systems [17]. Many studies have demonstrated that ginsenosides have various pharmacological effects, such as anti-cancer effect, anti-diabetic, pro-thrombotic, and anti-inflammatary activities [17,18,19,20]. Among them, compound K, one of the PTS ginsenosides, induces apoptosis and endoplasmic reticulum (ER) stress via STAT3 regulation in liver cancer [19]. In addition, combination treatments of ginsenoside Rg2 and Rh1 inhibited inflammation through suppressing lipopolysaccharide-induced toll-like receptor 4/NF-κB/STAT1 pathway in RAW264.7 cells [21]. One of the minor ginsenosides, ginsenoside-Rh1 (Rh1), is a metabolite of the PTS- types major ginsenosides Re and Rg1 [17,21]. Several studies have indicated that Rh1 inhibits cancer proliferation, migration, and invasion by regulating of MAPK or PI3K/Akt-mediated MMP1, 3, and 9 expressions in colorectal cancer, hepatocellular carcinoma, and astroglioma cells [22,23,24]. However, reports on the anti-cancer effects of Rh1 in TNBC and its molecular mechanisms have not been reported.

This study investigated the inhibitory effects of Rh1 on TNBC. Rh1 treatment significantly induced mitochondrial dysfunction-mediated ROS production, leading to the inhibition of the STAT/NF-κB signaling pathway in MDA-MB-231 cells.

## 2. Results

### 2.1. Rh1 Inhibited Cell Viability, Migration, and Invasion in MDA-MB-231 Cells

**Figure 1:**
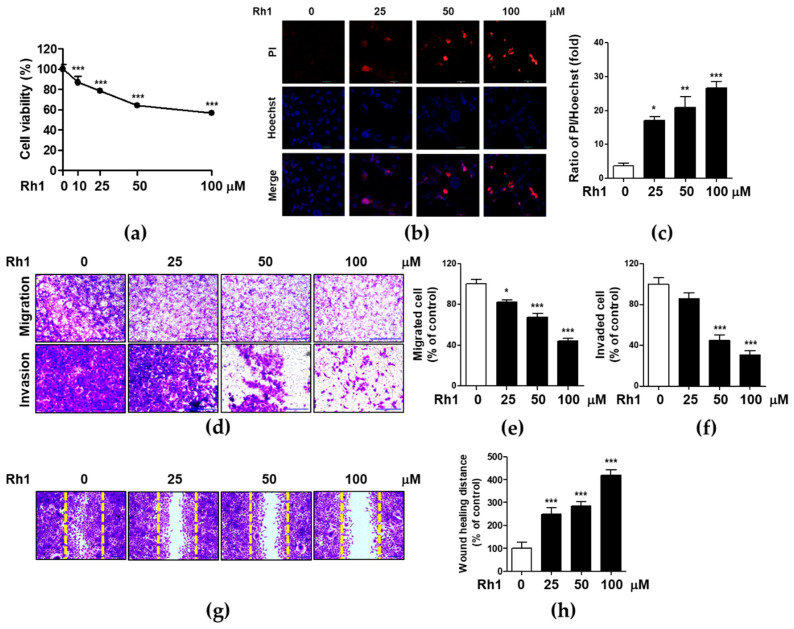
Rh1 inhibited cell viability, migration, and invasion in MDA-MB-231 cells (**a**) MDA-MB-231 cells were treated with indicated concentrations of Rh1 for 24 h. Cell viability was evaluated by MTT assay. (**b**,**c**) MDA-MB-231 cells were treated with Rh1 for 24 h. After dual stained with Hoechst 33324 and propidium iodide (PI), the cells were observed under a fluorescence microscope, and the numbers of dead cells were quantified. The cells stained with PI (red fluorescence) were indicated as dead cells. The scale bar indicates 30 μm. (**d**) Images indicating the migration (upper) and invasion (bottom) of MDA-MB-231 cells through polycarbonate membrane. The bar indicates 200 μm. (**e**,**f**) The number of cells were counted and shown in the column graph of the corresponding pictures. (**g**) After treatment with Rh1 for 36 h, cells were fixed, stained with crystal violets, and photographed using an Olympus microscope. The scale bar indicates 200 μm. (**h**) The migration area was measured using image J software and indicated as fold change compared to the control (0) sample. The data are presented as means ± SEM (*n* = 3). * *p* < 0.05, ** *p* < 0.01 or *** *p* < 0.001 compared with no treatment condition.

The cytotoxic effect of Rh1 on MDA-MB-231 cells was evaluated using MTT and propidium iodide (PI) staining assays. Cells were treated with different doses of Rh1 for 24 h. Rh1 significantly inhibited the viability of MDA-MB-231 (Figure 1a). PI staining indicates apoptotic cells by staining damaged nuclei. Rh1 significantly increased the number of apoptotic cells from 25 μM compared to non-treated cells (Figure 1b,c). Thereafter, we investigated the inhibitory effects of Rh1 on the migration and invasion of MDA-MB-231 cells. Rh1 treatment strongly suppressed cell migration and invasion in a dose-dependent manner (Figure 1d–f). In the invasion assay, Rh1 significantly suppressed the cell invasive capacity at 50 μM Rh1 (Figure 1d, bottom panel, and Figure 1f). Rh1 treatment significantly decreased wound healing ability in a dose-dependent manner in MDA-MB-231 cells (Figure 1g,h). These results indicate that Rh1 has a superior ability to regulate cancer cell wound healing, migration, and invasion in MDA-MB-231 cells.

### 2.2. Rh1 Inhibited Cell Migration via Inhibiting MMP2, MMP9, and VEGF-A

**Figure 2:**
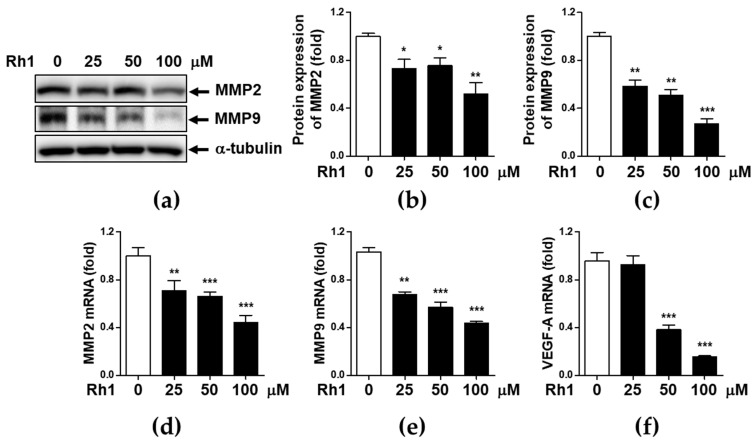
Rh1 inhibited cell migration via inhibiting MMP2, MMP9, and VEGF-A. (**a**–**c**) MDA-MB-231 cells were treated with 25, 50, and 100 μM of Rh1 for 12 h. Whole protein lysates were subjected to western blotting against MMP2 and MMP9 (**a**–**c**), and RNA samples were subjected to qPCR against MMP2, MMP9, and VEGF-A (**d**–**f**). The data are presented as means ± SEM (*n* = 3). * *p* < 0.05, ** *p* < 0.01 or *** *p* < 0.001 compared with each control.

To determine whether MMPs and their regulatory inhibitors are responsible for the inhibition of Rh1 on the metastatic potential of cancer cells, MDA-MB-231 cells were treated with 25, 50, and 100 μM Rh1 for 12 h. Thereafter, the expression of MMP2, MMP9, and VEGF-A was determined by western blot analysis and qRT-PCR. Rh1 treatment resulted in a dose-dependent reduction in MMP2 and MMP9 protein expression (Figure 2a–c). Consistent with the protein expression results, mRNA levels of MMP2, MMP9, and VEGF-A were strongly inhibited by Rh1 treatment in MDA-MB-231 cells (Figure 2d–f).

### 2.3. Rh1 Induced Mitochondrial Dysfunction via Producing Mitochondrial ROS

**Figure 3:**
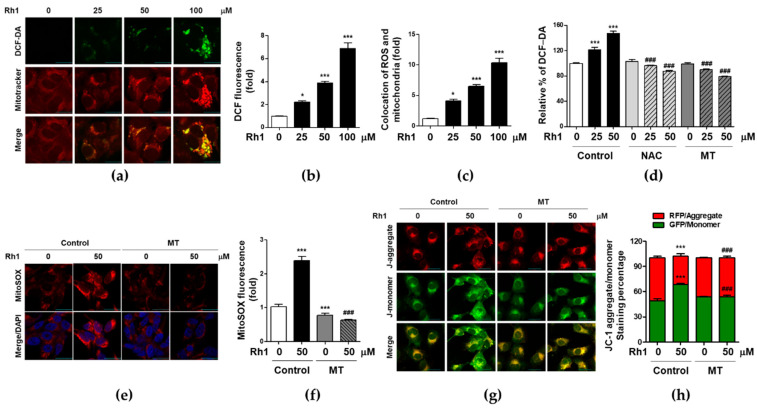
Rh1 induced mitochondrial dysfunction via producing mitochondrial ROS. (**a**–**c**) MDA-MB-231 cells were treated with the indicated doses of Rh1 for 24 h and intracellular ROS generation and morphology of mitochondria were determined by fluorescence assay. (**b**–**c**) The fluorescence intensity of DCF-DA (**b**) or mitotracker (**c**) was measured using image J software and indicated as fold change compared to the control (no treatment). The scale bar indicates 30 μm. (**d**) MDA-MB-231 cells were pretreated with 5 μM of mito-TEMPO (MT) or 10 mM NAC, followed by treatment with the indicated doses of Rh1 for 24 h and intracellular ROS generation was determined by DCF-DA assay. (**e**–**h**) MDA-MB-231 cells were pretreated with 5 μM of MT for 1 h followed by treatment with 50 μM Rh1 for 24 h. The fluorescence intensity of MitoSOX (**e**,**f**) or JC-1 (**g**,**h**) was measured using image J software and indicated as fold change compared to the control. The scale bar indicates 30 μm. The data are presented as means ± SEM (*n* = 3). * *p* < 0.05 or *** *p* < 0.001 vs control. ^###^ *p* < 0.001 compared with each inhibitor alone.

Intracellular ROS production has been reported to be related to various stresses. ROS affects cell apoptosis, migration, and invasion, which are generated by mitochondrial metabolism [25]. To elucidate whether Rh1 modulates the production of ROS in MDA-MB-231 cells, cells were treated with various concentrations of Rh1 for 24 h. Thereafter, ROS levels were analyzed by DCF-DA staining. Rh1 treatment significantly increased ROS accumulation in a dose-dependent manner in MDA-MB-231 cells (Figure 3a, upper panel, and Figure 3b). Next, we investigated mitochondrial morphological changes that divided fission and fusion (Figure 3a, middle panel). Mitochondrial fission plays an important role in disorder-associated processes, such as mitochondrial dysfunction, autophagy, and apoptosis. As shown in the Figure 3a middle panel, Rh1 increased the colonic shape, indicating mitochondrial fission. In addition, Rh1 induced ROS generation, which overlapped with morphological changes in mitochondrial fission (Figure 3c). In addition, Rh1-induced ROS production was inhibited by NAC, an ROS scavenger and Mito-TEMPO (MT), mtROS inhibitor (Figure 3d). MitoSox staining assay revealed that Rh1 significantly induced mtROS generation, whereas 5 μM MT treatment elicited the opposite effect (Figure 3e,f). Rh1-induced mtROS production was associated with mitochondrial damage as determined by the JC-1 assay (Figure 3g,h). Rh1 induced the fluorescence intensity of J-monomer in a dose-dependent manner, but MT treatment inhibited Rh1-induced mitochondrial membrane disruption. In summary, the results suggest that Rh1 induced apoptosis by the production of mtROS.

### 2.4. Mitochondrial ROS Is Associated with Rh1-Inhibited Cell Migration and Invasion

**Figure 4:**
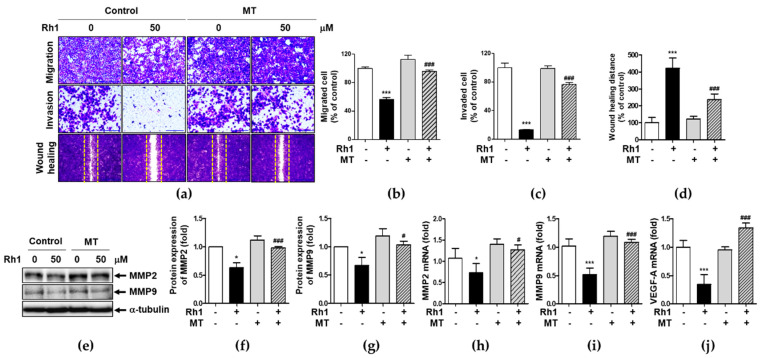
Mitochondrial ROS is associated with Rh1-inhibited cell migration and invasion. (**a**) MDA-MB-231 cells were pretreated with 5 μM MT for 1 h followed by treatment with 50 μM Rh1. After 36 h, cells were fixed, stained with crystal violet, and photographed using an Olympus microscope. Each Image indicates the migration (upper), invasion (middle), and wound healing activity (bottom) of MDA-MB-231 cells through polycarbonate membrane. The scale bar indicates 200 μm. (**b**–**d**). The number of cells were counted and shown in the column graph of the corresponding pictures. (**e**–**j**) MDA-MB-231 cells were pretreated with 5 μM MT for 1 h followed by treatment with 50 μM of Rh1 for 12 h and whole protein lysates and mRNA samples were subjected to western blotting against MMP2 and MMP9 (**e**–**g**) or qRT-PCR against MMP2, MMP9, and VEGF-A (**h**–**j**). The data are presented as means ± SEM (*n* = 3). * *p* < 0.05 or *** *p* < 0.001 compared with control. ^#^ *p* < 0.05 or ^###^ *p* < 0.001 compared with MT alone.

To determine the effect of Rh1-induced mtROS on cell migration and metastasis, cells were pretreated with 5 μM MT for 1 h followed by treatment with 50 μM Rh1. MT significantly suppressed Rh1-inhibited migration of MDA-MB-231 cells (Figure 4a, upper panel, Figure 4b,d). Consistent with the migration data, treatment with MT significantly sustained Rh1-inhibited invasion (Figure 4c). This suggests that Rh1 inhibited migration and invasion via the induction of mtROS production in MDA-MB-231 cells. In addition, Rh1 treatment inhibited expression of MMP2 and MMP9, whereas MT strongly suppressed Rh1 effects (Figure 4e–g). Consistently, Rh1-inhibited MMP2, MMP9, and VEGF-A expression was suppressed by the MT treatment (Figure 4h–j). These results show that Rh1 inhibited expression of MMP2, MMP9, and VEGF-A by the production of mtROS.

### 2.5. Rh1 Inhibited STAT3 Activation via Inducing mtROS

**Figure 5:**
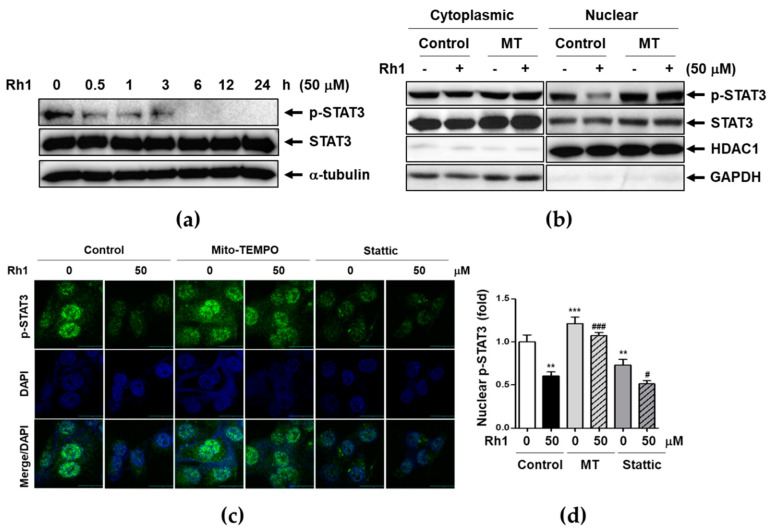
Rh1 inhibited STAT3 activation via inducing mtROS. (**a**) MDA-MB-231 cells were treated with 50 μM Rh1 for indicated time intervals. (**b**) MDA-MB-231 cells were pretreated with 5 μM MT for 1 h followed by treatment with 50 μM Rh1 for 30 min. After fractionation, protein localization was evaluated by western blot analysis. (**c**,**d**) MDA-MB-231 cells were pre-treated with 5 μM MT or 1 μM stattic followed by treatment with 50 μM Rh1 for 30 min. After treatment, cells were subjected to immunofluorescence staining, and p-STAT3 expression in cells was observed using a laser scanning confocal spectral microscope. The fluorescence intensity was measured using an Image J software and indicated as fold change compared to the control (0) sample. The scale bar indicates 30 μm. The data are presented as means ± SEM (*n* = 3). ** *p* < 0.01 or *** *p* < 0.001 vs. control. ^#^ *p* < 0.05 or ^###^ *p* < 0.001 compared with each inhibitor alone.

STAT3 has been shown to promote cancer proliferation, migration, invasion, and angiogenesis [26]. The aberrantly activated form of STAT3 promotes tumorigenesis in many cancers, including TNBC [9]. To investigate whether Rh1 modulated STAT3 activation, MDA-MB-231 cells were treated with 50 μM Rh1 for the indicated times, and STAT3 activation was examined by western blot analysis. We found that phosphorylation of STAT3 was dramatically suppressed by Rh1 for 30 min in a time-dependent manner (Figure 5a). Thereafter, we examined whether inhibition of Rh1-induced mtROS production was involved in p-STAT3 nuclear accumulation and STAT3 nuclear translocation. As shown in Figure 5b, MT-induced inhibition of basal mtROS levels caused STAT3 activation in the nucleus and STAT3 nuclear translocation compared to the control condition. Rh1 significantly inhibited the accumulation of p-STAT3 in the nucleus and STAT3 nuclear translocation, whereas the pretreatment with MT blocked these processes. We further confirmed the changes in p-STAT3 nuclear accumulation using an immunofluorescence assay (Figure 5c,d). Consistent with the western blot data, Rh1-inhibited p-STAT3 nuclear accumulation was recovered by MT treatment. Inhibition of p-STAT3 accumulation in the nucleus was confirmed by treatment with stattic, a small-molecule STAT3 inhibitor. In addition, the inhibitory effect of Rh1 on p-STAT3 accumulation in the nucleus was further inhibited by co-treatment with stattic (Figure 5c,d).

### 2.6. Rh1 Inhibits NF-κB Translocation and Transcriptional Activity through mtROS-induced STAT3 Deactivation

**Figure 6:**
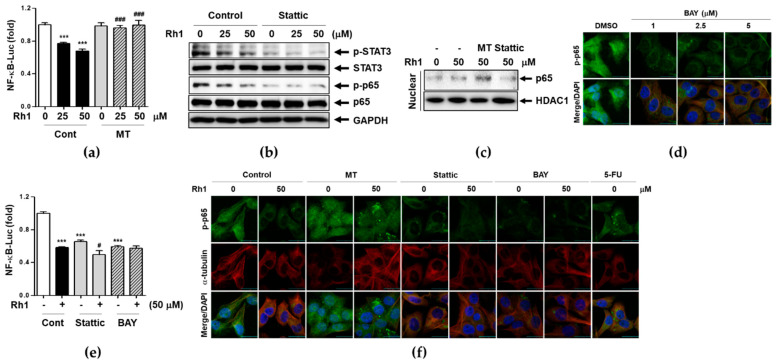
Rh1 inhibits transcriptional activity of NF-κB through STAT3 inactivation via production of mtROS. (**a**) MDA-MB-231 cells were co-transfected with pNF-κB-Luc and Renilla-Luc for 18 h. After transfection, cells were pretreated with 5 μM MT for 1 h followed by treatment with 25 and 50 μM Rh1. NF-κB-Luc promoter activity was measured using dual-luciferase reporter assay. Relative NF-κB-Luc promoter activity was determined and normalized by Renilla luciferase activity. (**b**) MDA-MB-231 cells were pretreated with 1 μM stattic for 1 h followed by treatment with 25 and 50 μM of Rh1 for 30 min. Whole cell lysates were subjected to western blotting against the indicated antibodies. (**c**) MDA-MB-231 cells were pretreated with 5 μM MT or 1 μM stattic followed by treatment with 50 μM Rh1 for 30 min. After fractionation, protein localization was evaluated using western blot analysis. (**d**) MDA-MB-231 cells were treated with 1, 2.5, or 5 μM BAY for 3 h and p-NF-κB expression in cells was observed using a laser scanning confocal spectral microscope. The scale bar indicates 30 μm. (**e**) MDA-MB-231 cells were co-transfected with pNF-κB-Luc and Renilla-Luc for 18 h. After transfection, cells were pretreated with 1 μM stattic or 1 μM BAY for 1 h followed by treatment with 50 μM Rh1 for 12 h. (**f**) MDA-MB-231 cells were pretreated with 5 μM MT, 1 μM stattic, or 1 μM BAY for 3 h followed by treatment with 50 μM Rh1 for 24 h. After treatment, cells were subjected to immunofluorescence staining, and p-p65 expression in cells was observed using a laser scanning confocal spectral microscope. The fluorescence intensity was measured using an Image J software and indicated as fold change compared to the control (0) sample. The scale bar indicates 30 μm. The data are presented as means ± SEM (*n* = 3). *** *p* < 0.001 vs control. ^#^ *p* < 0.05 or ^###^*p* < 0.001 compared with each inhibitor alone.

To investigate whether Rh1-mediated mtROS regulates NF-κB promoter activity, another major metastatic marker, MDA-MB-231 cells were co-transfected with NF-κB-Luc and pRL-TK plasmids and luciferase activity in the cell extracts was using a dual-luciferase reporter assay system. As shown in Figure 6a, Rh1 significantly inhibited NF-κB-Luc activity in a dose-dependent manner, whereas MT treatment significantly inhibited Rh1’s effects on NF-κB-Luc activity (Figure 6a). A previous report suggested that p-STAT3 can bind to p65, one of the subunits of NF-κB to promote the NF-κB-mediated signaling pathway [27]. To investigate the relationship between STAT3 and NF-κB activation, cells were pretreated with 1 μM stattic for 1 h followed by treatment with 25 and 50 μM Rh1 for 30 min. Rh1 inhibited both STAT3 and p65 phosphorylation, and stattic co-treatment further decreased both protein activation (Figure 6b). When mtROS was blocked by MT treatment, the inhibitory effect of Rh1 on p65 translocation into nucleus was reversed, whereas stattic treatment further inhibited p65 translocation (Figure 6c), suggesting the role of mtROS-deactivated STAT3 in p65 translocation. P-p65 NF-κB translocation was examined by immunofluorescence assay after treatment with BAY and found that BAY completely blocked p65 NF-κB translocation into the nucleus at a concentration 1 μM (Figure 6d). Interestingly, inhibition of STAT3 or NF-κB activation by stattic or BAY 11-7082 (BAY) induced the deactivation of NF-κB-Luc as much as Rh1 treatment (Figure 6e). We confirmed the effect of Rh1-induced mtROS and STAT3 deactivation on p-p65 translocation using immunofluorescence assay. Consistent with the results shown in Figure 6c, p-p65 translocation was completely blocked by Rh1 co-treatment with stattic or BAY, whereas MT pretreatment abolished Rh1’s effects on p-p65 translocation (Figure 6f).

### 2.7. Rh1 Inhibits Cell Migration via Down-Regulating STAT3/NF-κB Signaling Pathway

**Figure 7:**
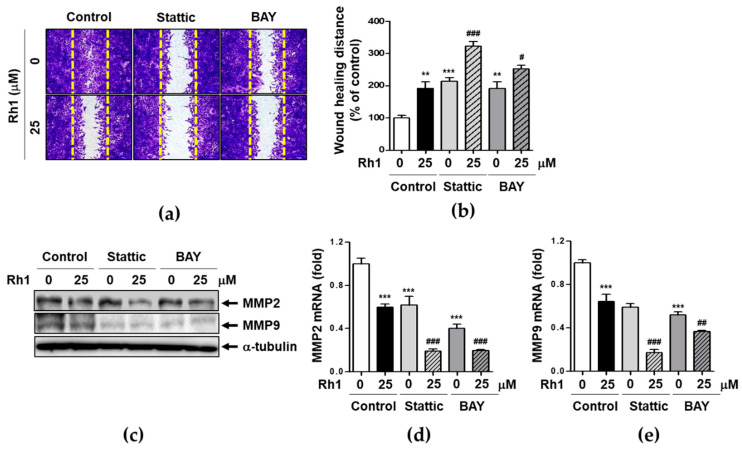
Rh1 inhibits cell migration via down-regulating STAT3/NF-κB signaling pathway. (**a**,**b**) MDA-MB-231 cells were grown as a nearly confluent monolayer culture and then scratched using pipette tip. Cells were pretreated with 0.5 μM stattic or 1 μM BAY for 1 h followed by treatment with 25 μM Rh1 for 36 h. After 36 h, cells were fixed, stained with crystal violet, and photographed using an Olympus microscope. The scale bar indicates 200 μm. (**b**) The migration area was measured using Image J software and indicated as fold changes compared to control (0) sample. (**c**) MDA-MB-231 cells were pretreated with 0.5 μM stattic and 1μM BAY for 1 h followed by treatment with 25 μM Rh1 for 12 h and whole protein lysates and RNA samples were subjected to western blotting against MMP2 and MMP9 or qRT-PCR (**d**,**e**) against MMP2 and MMP9. The data are presented as means ± SEM (*n* = 3). ** *p* < 0.01 or *** *p* < 0.001 compared with control. ^#^ *p* < 0.05, ^##^ *p* < 0.01 or ^###^ *p* < 0.001 compared with each inhibitor alone.

To determine the role of STAT3 and NF-κB inactivation in Rh1-treated MDA-MB-231 cell metastasis, cells were pretreated with 0.5 μM of stattic or BAY (1 μM) for 1 h followed by treatment with 25 μM of Rh1. Low concentrations of each compound were chosen to evaluate the synergic effects of STAT3/NF-κB signaling regulation and Rh1 in the experiment. As shown in Figure 7a,b, Stattic or BAY treatment significantly suppressed the wound healing activity. In particular, co-treatment of stattic or BAY with Rh1 synergistically reduced wound healing activity compared to Rh1. Mechanistically, inactivation of STAT3 and NF-κB with each inhibitor and Rh1 significantly inhibited both MMP2 and MMP9 protein expression (Figure 7c). Furthermore, mRNA levels of MMP2 and MMP9 were inhibited by Rh1, Stattic, or BAY (Figure 7d,e). Consistent with the protein expression data, combination treatment with each inhibitor and Rh1 more significantly inhibited mRNA levels of MMP2 and MMP9 than Rh1 alone (Figure 7d,e).

Our data demonstrated that STAT3 and NF-κB are involved in MDA-MB-231 cell metastasis, and combination of Rh1 and stattic or BAY, strongly inhibited metastasis in MDA-MB-231 cells.

## 3. Discussion

Although the prognosis and survival of BC patients have increased with the development of BC therapeutic strategies, metastasis is the most common cause of cancer death in approximately 90% of BC patients [1]. Through the invasion-metastasis cascade, cancer cells can move to other organs such as, lung, bone marrow, and brain, leading to cancer-derived deaths [3]. Many studies have indicated that bioactive compounds can inhibit BC metastasis by targeting various signaling pathways, including EGFR and STAT, and regulation of ROS production [9,28,29]. Among them, 20(S)-protopanaxadiol inhibited EGFR/MAPK signaling pathway-mediated BC migration, invasion, and epithelial-mesenchymal transition [30]. Rh1 is one of the minor ginsenosides and metabolites of Re and Rg1 formed by the intestinal microbiota [23]. Rh1 has various pharmacological effects, including anticancer, anti-inflammatory, and antibiotic-effect [21,22,31]. However, the effects of Rh1 on metastasis have not been elucidated in TNBC. In this study, we investigated the effects of Rh1 on metastasis in MDA-MB-231 cells and the underlying signaling pathways.

First, we found that Rh1 displayed anticancer effects by inhibiting cell viability, wound healing potential, migration, and invasion in a dose-dependent manner in MDA-MB-231 cells (Figure 1). Rh1 inhibited migration molecules, such as MMP2 and MMP9, and the angiogenesis molecule VEGF-A expression at the mRNA and protein levels (Figure 2). Rh1 inhibited the expression of MMP9 and VEGF-A more than MMP2 in the 50 μM Rh1 treatment. In fact, Rh1 has been reported to exhibit low plasma concentrations after oral administration due to its low oral bioavailability [32]. However, plasma detection was hard because of rapid metabolism in the intestine and Rg1, a precursor of Rh1, showed anti-cancer and anti-inflammatory effect by converting to Rh1 in the intestine [33]. Therefore, the plasma concentration of Rh1 and its pharmacokinetic-pharmacodynamic relationship needs to be further investigated in the future study.

Many studies have shown that ROS production is involved in BC metastasis through several many mechanisms, such as mitochondrial dysfunction, MAPK, and PI3K/Akt signaling pathways [9,14,29]. A previous study reported that *Taraxacum afficinale* extract isolated from dandelion suppressed migration and invasion by reducing mitochondrial integrity in neuroblastoma [34]. In our study, Rh1 increased mtROS production, which induced mitochondrial disruption (Figure 3). In addition, MT treatment significantly suppressed Rh1-induced inhibition of wound healing activity, migration, and invasion by sustained expression of MMP2, MMP9, and VEGF-A in MDA-MB-231 cells (Figure 4).

STAT3 signaling pathway is a key molecular mechanism involved in a variety of cancer metastasis and tumorigenesis [12,19,28]. A previous study reported that excessive ROS production induces cancer apoptosis via inhibition of STAT3-mediated ER stress activation in prostate cancer and BC cells [8,35,36]. Our data showed that Rh1 decreased nuclear expression of p-STAT3, whereas MT treatments significantly accumulated nuclear p-STAT3 (Figure 5c). It suggests that Rh1-induced mtROS production is involved in the inhibition of STAT3 activation (Figure 5).

It has been reported that activation of STAT3 promotes cell migration and invasion, which is linked to the induction of MMP2 and MMP9 expression or interleukin-22 expression [37]. Mechanistically, STAT3 promotes TNBC progression by cross-linking with phosphorylation of NF-κB p65, leading to its signaling pathway associated with metastatic gene expression of cancer or immune cells [38]. NF-κB or STAT3 promoter activation was increased gene expression of MMPs that signals induce cancer metastasis [39]. Consistent with previous reports, we demonstrated that treatment with Rh1 significantly suppressed p-STAT3 nuclear accumulation and affected NF-κB p65 nuclear localization and promoter activity (Figure 5 and Figure 6). Inhibition of STAT3 activity by stattic treatments suppressed phosphorylation of NF-κB p65, which was decreased by Rh1 treatments (Figure 6b). In addition, Rh1 suppressed nuclear translocation of p-p65, whereas inhibition of STAT3 inhibited p-p65 translocation as much as BAY, a specific inhibitor of NF-κB activation (Figure 6f). Furthermore, Rh1-inhibited NF-κB-Luc activity was completely reversed by stattic or BAY treatment (Figure 6a). In this process, Rh1-induced mtROS production was involved in the inhibition of STAT3 and NF-κB activation leading to the suppression of cell metastatic functions through NF-κB transcriptional targets, MMP2 and MMP9 expression (Figure 7).

**Figure 8:**
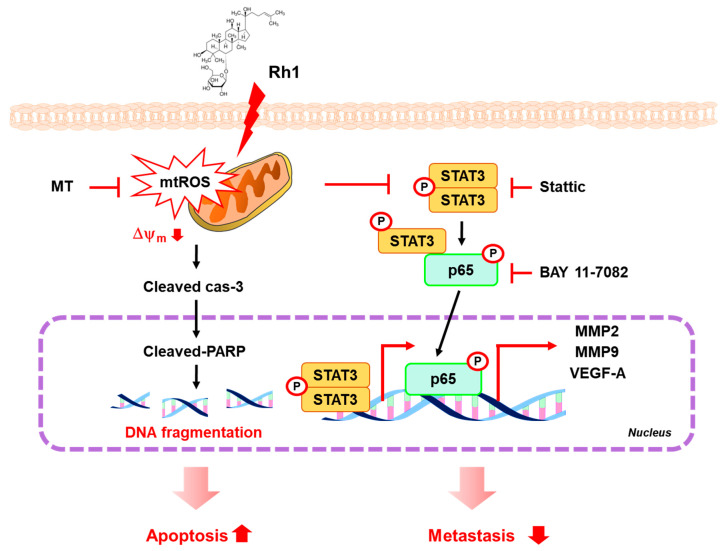
A schematic showing the signaling pathways mediated the inhibitory effect of Rh1 on MDA-MB-231 cell migration and invasion. Rh1 inhibits viability, migration, and invasion through inhibition of MMP2, MMP9, and VEGF-A. Treatment with Rh1 significantly increased mitochondria damage-induced mtROS production, which was inhibited by MT. In addition, inhibition of mtROS production by MT treatment, suppressed Rh1-inhibited migration and invasion through regulating gene expression of MMP2, MMP9, and VEGF-A. Rh1 inhibited phosphorylation of STAT3. STAT3 also contributed nuclear p65 expression, which was inhibited by stattic treatment. In addition, Rh1 decreased STAT3 activation-mediated NF-κB transcriptional activity. Moreover, treatment with Rh1 synergistically enhanced the effects of STAT3 and NF-κB inhibition by suppressing of MMP2, MMP9, and VEGF-A expression in MDA-MB-231 cells. Ginsenoside-Rh1, Rh1; IκBα, NF-κB inhibitor alpha; MMP, matrix metalloproteinase; MT, Mito-TEMPO; mtROS, mitochondria ROS; p65, protein 65; VEGF-A, vascular endothelial growth factor-A; Δψm, mitochondria membrane potential.

In conclusion, treatment with Rh1 significantly induced mitochondrial dysfunction through increasing mtROS production, leading to the inhibition of the STAT/NF-κB signaling pathway in MDA-MB-231 cells. In addition, Rh1 inhibited migration, invasion, and angiogenesis by suppressing the STAT3/NF-κB signaling pathway (Figure 8). Therefore, Rh1 may serve as a potential candidate for the inhibition of TNBC metastasis.

### 3.1. Materials

Rabbit anti-phospho p65 Ser 536, rabbit anti-phospho STAT3 Tyr705, and rabbit anti-STAT3 antibodies were purchased from Cell Signaling Technology, Inc. (Danvers, MA, USA). Mouse anti-α-tubulin and mouse anti-NF-κB antibodies were purchased from Santa Cruz Biotechnology (Paso Robles, CA, USA). Rabbit anti-MMP2, rabbit anti-MMP9, and skim milk (1.1363) were purchased from Merck millipore. (Burlington, MA, USA). 10X phosphate buffered saline (PBS, #EBA-1105) and reveres transcription 5X master mix (#EBT-1511) were purchased from ELPIS-BIOTECH (Daejeon, South Korea). Tri-RNA reagent (#FATRR-001) was purchased from Favorgen (Pingtung, China). Ammonium persulfate (#A3678), 2-mercaptoethanol (#M6250), 2′,7′-Dichlorofluorescin diacetate (DCF-DA, #D6883), MT (SML0737), and Rh1 (#56805) were purchased from Sigma-Aldrich (St. Louis, MO, USA). Clarity western ECL substrate (#170-5061) and iQ SYBR green supermix (#170-8882AP) were purchased for BIO-RAD (Hércules, CA, USA). EzReprobe (#WSE-7240) and EzRIPA Lysis kit (#WSE-7240) were purchased for ATTO corporation (Tokyo, Japan). 0.25% Trypsin/EDTA (#25200-072) was purchased from Gibco (Waltham, MA, USA).

### 3.2. Cell Culture and Viability Assay

MDA-MB-231 human breast cancer cells were obtained from the American Type Culture Collection (Manassas, VA, USA). Cell viability was determined using 3-(4,5-dimethylthiazole-2-yl)-2, 5-diphenyltetrazolium bromide assay (MTT, Sigma-Aldrich) according to our previous report [12]. For the assay, cells were seeded in 96-well at a density of 1 × 10^4^ cells/well. After 4 h of stabilization, cells were treated with various doses of Rh1 for 24 h followed by treatment with MTT (12 mM) in FBS-free DMEM media for 2 h at 37 °C with 5% CO_2_. The medium was the removed and the precipitated formazan was extracted in DMSO. The absorbance was measured at 540 nm using a microplate reader (TECAN, Männedorf, Switzerland).

### 3.3. PI Staining Assay

Effects of Rh1 on cell apoptosis and cell death were evaluated by detecting the fluorescent intensity of cells stained with PI (Sigma, 537059). MDA-MB-231 cells were seeded in a 12-well plate at the 5 × 10^5^ cells/well. Cells were starved for 4 h in the serum-free DMEM. Then the cells were treated with 0, 25, 50, and 100 μM Rh1 for 24 h. Next, the cells were thoroughly incubated in 10 ug/mL PI for 30 min at 37 °C and 5% CO_2_. Representative images were automatically taken using a SPOT digital camera. Quantitative analysis was performed using Image J.

### 3.4. Western Blot

Cells were washed with PBS and lysates were prepared using radioimmunoprecipitation (RIPA) buffer including 50 mM Tris-HCl [pH 7.4], 150 mM NaCl, 1 mM EDTA, 1% Nonidet P-40, 0.1% SDS, 1 mM dithiothreitol, 1:200-diluted protease inhibitor cocktail (ATTO), 1 mM PMSF and 10 mM NEM, and 0.1 mM iodoacetamide. The protein extracts were resolved by SDS-PAGE, electro transferred onto a PVDF membrane, and visualized by using the clarity western ECL substrate (BIO-RAD) according to the manufacturer’s instructions. Each protein level was detected by western blotting with each corresponding specific antibody.

### 3.5. Quantitative Real-Time Polymerase Chain Reaction Assay

The quantitative RT-PCR (qRT-PCR) assay was used to analyze the mRNA expression of MMP2, MMP9, and VEGF-A as described previously [12]. The relative gene expression was calculated using a 2^−∆ct^ method, which was normalized by GAPDH. All primer sequences used in qRT-PCR experiments are listed as following. MMP2; Forward-5′-ACAAAGAGTTGGCAGTGCAATA-3′, Reverse-5′-TCTGGTCAAGATCACCTGTCTG-3′, MMP9; Forward-5′-CAGTCCACCCTTGTGCTCTT -3′, Reverse-5′-CCAGAGATTTCGACTCTCCAC-3′, VEGF-A; Forward-5′-ACATCTTCAAGCCATCCTGTG-3′, Reverse-5′-TGTTGTGCTGTAGGAAGCTCAT-3′, GAPDH; Forward-5′-GCACCGTCAAGGGCTGAGAAC -3′, Reverse-5′-TGGTGAAGACGCCAGTGGA-3′.

### 3.6. In Vitro Wound-Healing Assay

The wound healing assay was used to analyze the potential of cancer wound healing as described our previous reports [12]. MDA-MB-231 cells were seeded in 6-well plate at a density of 1 × 10^6^ cells/well. After 24 h, the monolayers were scratched with a 200 μL pipette tip for creating a wound area and washed twice with serum-free media. Cells were treated with Rh1 for 36 h. The rate of wound closure was assessed and imaged. Each image is derived from the five randomly selected fields. Images were acquired using an Olympus BX51 equipped with a DP72 digital microscope camera (Olympus Cooperation, South Korea). Quantitative analysis was performed using Image J.

### 3.7. Transwell Migration and Invasion Assay

Transwell plate (Corning, NY, USA) was used to analyze cell migration and invasion ability. A total of 1 × 10^5^ cells were seeded on the transwell chamber and 200 μL of serum-free media was added. The lower compartment was filled with 600 μL of culture medium. After 18 h, the upper chambers with the residual cells were removed and the cells under the surface were fixed with 4% NPA and then stained cell with 0.25% crystal violet for 10 min. Each image was derived from the five randomly selected fields. Images were acquired using an Olympus BX51 equipped with a DP72 digital microscope camera (Olympus Cooperation, South Korea). For transwell invasion assay, chambers pre-coated with matrigel (20 μL/well, 1.2 mg/mL) at 37 °C for 2 h were used. The similar protocol was followed as an above-mentioned migration assay.

### 3.8. Measurement of Mitochondria Activity

Mitochondrial activity was measured by detecting the fluorescent intensity of cells stained with mitotracker (Thermofisher scientific, M7513). MDA-MB-231 cells were seeded in 12-well plate at the 5 × 10^5^ cells/well. Cells were starved for 4 h in the serum free DMEM. After treatment, cells were thoroughly incubated in 500 nM mitotracker for 30 min at 37 °C and 5% CO_2_. Representative images were automatically taken using a SPOT digital camera. Quantitative analysis was performed using Image J.

### 3.9. Measurement of Mitochondria-Derived ROS

Mitochondria-derived ROS production was measured by detecting the fluorescent intensity of cells stained with mitoSOX (Thermofisher scientific, M36008). MDA-MB-231 cells were seeded in 12-well plate at the 5 × 10^5^ cells/well. Cells were starved for 4 h in the serum-free DMEM. Then the cells were pretreated with 5 μM MT for 1 h followed by treatment with 25, 50, and 100 μM Rh1 for 24 h. Next, the cells were thoroughly incubated in 5 μM mitoSOX for 10 min at 37 °C and 5% CO_2_. After staining, cells were fixed by using 4% paraformaldehyde. Representative images were automatically taken using a SPOT digital camera. Quantitative analysis was performed using Image J.

### 3.10. Measurement of Intracellular ROS Production

Intracellular ROS levels were measured by detecting the fluorescent intensity of cells stained with 2′,7′-dichlorodihydrofluoresceindiacetate (DCF-DA) assay as described in our previous report [40]. MDA-MB-231 cells were seeded in 96-well plate at the 1 × 10^4^ cells/well. Cells were starved for 4 h in the serum-free DMEM. Then the cells were treated with 25, 50, and 100 μM of Rh1 for 24 h. Next, the cells were thoroughly washed with PBS and incubated in 10 μM DCF-DA for 30 min at 37 °C and 5% CO_2_. Cells were washed with PBS and the fluorescence intensity of DCF was measured by using the fluorescence microplate reader with an excitation wavelength of 485 nm and an emission wavelength of 530 nm (TECAN, Männedorf, Switzerland). For quantifying fluorescence intensity of DCF-DA, pictures were taken randomly from 5 areas of each sample. After taking pictures, fluorescence intensity was measured by image J software. Next, we calculated expression of fluorescence by fold change compared with no treatment condition.

### 3.11. Luciferase Reporter Assay

Cells were co-transfected with pNF-κB-Luc and Renilla-Luc plasmid by the Solfect (Biosolyx, Daegu, South Korea) followed by manufacture method [12]. After transfection, cells were pre-treated with each inhibitor for 1 h followed by treatment with indicated doses of Rh1 for 12 h. NF-κB promoter luciferase activity was assayed using a dual-luciferase reporter assay system.

### 3.12. Preparation of Cytosolic Extracts and Nuclear Extracts

The MDA-MB-231 cells were pretreated with 5 μM MT followed by treatment with 50 μM Rh1 for 30 min. Cytosolic and nuclear proteins were prepared as previously described [21]. The extracts were subjected to western blotting.

### 3.13. Immunofluorescence Assay and Confocal Laser-Scanning Microscopy

The effects of Rh1 on the expression of p-STAT3 and p-p65 was determined using immunofluorescence assay as described previously [41]. MDA-MB-231 cells were fixed with 4% paraformaldehyde for 10 min at RT and permeabilized with 0.1% Triton X-100 for 10 min. Cells were incubated with the primary antibody for 2 h at RT followed by incubation with anti-mouse secondary antibodies (Invitrogen, Carlsbad, CA, USA) conjugated with Alexa 488 at a dilution of 1:1000 for 1 h at room temperature. Cell nuclei were counter-stained with 40, 6-diamidino-2-phenylindol (DAPI) for 5 min. Slides were mounted with prolong gold antifade mount reagent (#P36930, Invitrogen) and examined with a laser scanning confocal spectral microscope (Nanoscope systems, South Korea). Representative images were automatically taken using a SPOT digital camera. Quantitative analysis was performed using Image J.

### 3.14. JC-1 Mitochondrial Membrane Potential Assay

The effects of Rh1 on mitochondria membrane potential were detected by the fluorescent intensity of cells with JC-1 staining. MDA-MB-231 cells were seeded in 12-well plate at the 5 × 10^5^ cells/well. Cells were starved for 4 h in the serum-free DMEM. Then the cells were pre-treated with 5 μM MT for 1 h followed by treatment with 25 and 50 μM Rh1 for 24 h. Next, cells were thoroughly incubated in 10 μg/mL JC-1 (420200, Sigma) for 20 min at 37 °C with 5% CO_2_. Fluorescence intensity of Δψm was detected under different condition (Ex (λ) 485 nm, Em (λ) 530 nm for monomer; Ex (λ) 510 nm, Em (λ) 560nm for aggregates) on the fluorescence microplate reader (TECAN, Männedorf, Switzerland). Representative images were automatically taken using a SPOT digital camera. Quantitative analysis was performed using Image J.

### 3.15. Statistical Analysis

Statistical analysis was performed using GraphPad Prism 5 (version 5.02, GraphPad Software Inc., San Diego, CA, USA). One-way analysis of variance (ANOVA) followed by a Bonferroni multiple comparison was performed. A *p* value < 0.05 was considered significant. Student’s *t*-test (for normally distributed data) was used for two group comparisons. All experiments were expressed as the mean ±SEM and were performed independently at least three times.

## Data Availability

The data presented in this study are available within the article text and figures.

## Abbreviations

BC	Breast cancer
BAY	BAY 11-7082
DAPI	40, 6-diamidino-2-phenylindol
DCF-DA	2′,7′-dichlorodihydrofluoresceindiacetate
MAPK	Mitogen-activated protein kinase
MMP	Matrix metalloproteinase
MT	Mito-TEMPO
mtROS	Mitochondria ROS
NAC	N-acetyl-cysteine
NF-κB	Nuclear factor kappa-light-chain-enhancer of activated B cells
PI3K/Akt	Phosphatidylinositol-3-kinase/protein kinase B
PTS	20(S)-protopanaxatriol saponins
Rh1	Ginsenoside Rh1
ROS	Reactive oxygen species
STAT	Signal transducer and activator of transcription
TNBC	Triple negative breast cancer
VEGF	Vascular endothelial growth factor
